# Supplementary material for: Population genomics and molecular epidemiology of wheat powdery mildew in Europe
Source: PLoS Biol. 2025 May 2;23(5):e3003097. doi: 10.1371/journal.pbio.3003097 (PMC12047814; doi:10.1371/journal.pbio.3003097)
Supplement: Attachment — Submitted filename: plos_biol_response_to_reviewers.pdf [file pbio.3003097.s006.pdf]

We would like to thank the two reviewers for reading our manuscript and for their feedback. We addressed all suggestions and concerns, and you find below our point-by-point response to all comments. We have numbered the reviewers' comments, and we have used a blue font for our responses. Line numbers refer to the 'track-changes' version of the revised manuscript.

Reviewer #1:

Overall, the manuscript "Population genomics and molecular epidemiology of wheat powdery mildew in Europe" uses genome sequencing data to provide valuable insights into the epidemiological dynamics of Blumeria graminis f.sp tritici in Europe and globally. The study effectively addresses key biological questions and employs a suitable methodology, leading to interesting and credible conclusions. I am listing below some -rather minor - comments and suggestions aimed at improving the manuscript.

1) Diversity:

Nucleotide diversity estimates reported in this manuscript are higher than those reported by Storipoulos et al. (ref 37), with a partially similar dataset.

why such differences? the reader must know why

The genome-wide summary statistics pi, theta and Tajima's D were computed for the *Europe+_recent* dataset. Only four of the isolates used in Sotiropoulos et al 2022 were sampled in Europe after 2015 and were thus included in the *Europe+_recent* dataset (which includes 368 samples). The datasets of the two studies are therefore not comparable. We have now added this explanation to the manuscript (Lines 844-847).

Further, Sotiropoulos et al used PopGenome to compute nucleotide diversity estimates. This software is known to output downwardly biased estimates in the presence of missing data (Pfeifer et al 2014; Korunes and Samuk 2021). For this reason, in our study, we used pixy (Korunes and Samuk 2021) to compute pi, which does not assume missing data to be invariant.

2) Shared ancestry:

The authors tend to over-interpret shared ancestry as evidence for admixture.

S1 appendix Page 36: shared ancestry is interpreted as admixture. However, shared ancestry is not necessarily caused by admixture. The authors should be more cautious. See figure 2 in Lawson et al. doi: 10.1038/s41467-018-05257-7

L143: Lawson et al. (cited above) show that shared ancestry is not necessarily a hallmark of admixture, especially if the age of samples or the sampling effort are variable: « Because STRUCTURE/ADMIXTURE accounts for the most salient variation, results are greatly affected by sample size in common with other methods. Specifically, groups that contain fewer samples or have undergone little population-specific drift of their own are likely to be fit as mixes of multiple drifted groups, rather than assigned to their own ancestral population. Indeed, if an ancient sample is put into a data set of modern individuals, the ancient sample is typically represented as an admixture of the modern populations, which can happen even if the individual sample is older than the split date of the modern populations and thus cannot be admixed. »

We agree with the reviewer that shared ancestry should not be interpreted strictly as admixture, as there are other scenarios without admixture events that could generate the same pattern. This was not our interpretation, but our text was ambiguous. We did write this in the original manuscript:

"One limitation common to both ADMIXTURE and fineSTRUCTURE is that they model genetic diversity in terms of discrete ancestries or populations. Therefore, these might not be the best tools to investigate gradients of diversity, or continuous patterns of differentiation"

We also wrote this in the original S1 Appendix:

"These assumptions are violated when there are gradients of diversity over space, such as those typical of isolation by distance. It has been shown before that methods such as

ADMIXTURE can infer multiple ancestries and admixed individuals to account for this variability".

We now updated the manuscript and S1 Appendix to clarify this point. In the revised version we do not interpret shared ancestry as evidence for gene flow (Lines 212-213, S1 Appendix Page 36: second paragraph of section "Population structure in Europe and the Mediterranean"). In addition, we added the following two sentences respectively to the manuscript and to S1 Appendix:

"Moreover, it was shown before that patterns of shared ancestry can also be generated by sampling biases or demographic processes that do not include admixture (Lawson et al 2018)."(Lines 222-224)

"Moreover, patterns of shared ancestry can also be generated by sampling biases or demographic processes that do not include admixture events (Lawson et al 2018)."

Isolation-by-distance:

3) It is unclear why the authors didn't use partial Mantel tests, i.e. correlation tests between two matrices, but controlling for the effect of a third distance matrix.

Partial mantel tests have been previously criticized for having inflated type-I error rates and the validity of their statistical assumptions in biological settings has been questioned (Raufaste and Rousset 2001, Legendre and Fortin 2010, Guillot and Rousset 2013). For these reasons, we refrain from using partial Mantel tests in the manuscript.

Nevertheless, as suggested by the reviewer, we performed simple and partial mantel tests on our data using the R package vegan. The results are reported in the table below:

Dataset	Test	Mantel correlation	Mantel p-value	Conditioned on	Partial Mantel correlation	Partial Mantel p-value
Europe+_2022_2023	Isolation by distance	0.472	0.001	Wind	0.079	0.005
				Climate	0.363	0.001
	Isolation by wind	0.547	0.001	Geography	0.323	0.001
				Climate	0.472	0.001
	Isolation by climate	0.33	0.001	Geography	0.066	0.075
				Wind	0.1	0.007
N_EUR_2022_2023	Isolation by distance	0.085	0.014	Wind	0.001	0.494
				Climate	0.178	0.001
	Isolation by wind	0.129	0.007	Geography	0.098	0.033
				Climate	0.159	0.004
	Isolation by climate	-0.049	0.883	Geography	-0.1645	0.998
				Wind	-0.106	0.982
S_EUR2_2022_2023	Isolation by distance	0.389	0.001	Wind	0.323	0.001
				Climate	0.254	0.001
	Isolation by wind	0.259	0.001	Geography	-0.124	0.998
				Climate	0.091	0.055
	Isolation by climate	0.342	0.001	Geography	0.164	0.001
				Wind	0.248	0.001

The main takeaways from this analysis are the same as those already reported in our manuscript (Lines 334-337): "We found that wind-distances had the highest correlation with genetic distances in the N_EUR population and overall, while geographic and climatic distances had a higher correlation with genetic distances in S_EUR2."

4) Fig 2 and corresponding text: the y-axis should be an estimate of identify-by-descent, and it's unclear whether the computed Hamming distance is a good estimate of identify-by-descent. Genetic relatedness (computed with PLINK or Kinship2) is an estimate of identify-by-descent.

See for instance doi: 10.1371/journal.pgen.1006911

We disagree with the reviewer. Genetic relatedness is not the only measure of genetic distance that can be used for isolation by distance analyses. For example, several studies used F_{ST} as a measure of genetic differentiation between populations (Angst et al 2022, Sønstebø et al 2022, Li et al 2023). Furthermore, previous studies focusing on different crop pathogens such as wheat powdery mildew (Sotiropoulos et al 2022), *Zymoseptoria tritici* (Feurtey et al 2023), and *Pyrenophora teres* (Taliadoros et al 2024) used the Hamming distance (or closely related measures) between individuals to perform isolation by distance analyses, similar to our approach.

In the study mentioned by the reviewer (Aguillon et al 2017), relatedness was used to "*explore the role of limited dispersal in shaping patterns of isolation-by-distance [...] at an exceedingly fine spatial scale (within ~10 km)*".

This is clearly different from our study, in which we sampled populations over thousands of kilometers. At this scale, sampled individuals are only distantly related, and measures of identity-by-descent cannot discern differences in relatedness beyond a few generations. Therefore, we consider Hamming distances a more appropriate choice for isolation-by-distance analyses for our dataset.

5) L211: please don't spread the (false) idea that small things can travel far, blown by the wind. Many fungal plant pathogens produce very small propagules that do not travel well with wind.

We eliminated any reference to the size of conidiospores, the sentence now reads: "Wind is considered as the main dispersal agent of powdery mildew. Conidiospores are the clonal propagules of Bgt, and it was shown that they can be transported by wind for hundreds of kilometers before landing on a new host". (Lines 313-316)

Avirulence gene AvrPm17:

6) Can the authors comment on the lack of loss-of-function mutations at AvrPm17? This is very surprising. If losing AvrPm17 allows overcoming the resistance conveyed by Pm17, one would expect a higher frequency of loss-of-function mutations.

This is a relatively common pattern for Bgt avirulence genes. A lack of loss-of-function mutations was reported before for *AvrPm3a2/f2*, *AvrPm3b2/c2*, and *AvrPm3d*. These observations are usually interpreted as evidence that avirulence genes have an important virulence function, i.e., that wheat powdery mildew needs these for a successful infection (Bourras et al 2019).

We now comment on this in the manuscript. (Lines 448-451)

7) Tajima's D (L604)

Tajima's D was calculated from Pi, estimated with pixy, and Theta, estimated with VCFtools.

However, VCFtools is notorious for providing wrong estimates of population genetic parameters because it typically assumes that sites with missing data have the same genotype as the reference.

Can the author check whether their estimates of Tajima's D are correct? This could be done, for instance, by computing Tajima's D or Watterson's Theta using the Scitkit-allel package.

This is a misunderstanding; we did not estimate Watterson's theta with VCFtools. VCFtools was used to count the number of biallelic segregating sites (S) in windows. These are simple counts and are not affected by the assumption about the genotypes of samples with missing data. Theta was subsequently calculated using the formula S/a1, where a1 is the $(n-1)^{th}$ harmonic number and n is the sample size. We then normalized this by the number of sites with at least one valid genotype in the window. We have rewritten this part in the methods section to describe our approach more clearly. (Lines 851-857)

Nonetheless, as suggested by the reviewer, we used scikit-allel to compute theta and verify the accuracy of our estimates. We found that the scikit-allel estimates are in the same range, though slightly lower than our estimates. This is expected, as scikit-allel uses a normalization factor equal to the window size (10000), while our normalisation factor included only sites with at least one valid genotype. Indeed, if we do not filter out sites without valid genotypes, we obtain the same estimate as scikit-allel, as seen in the figure below.

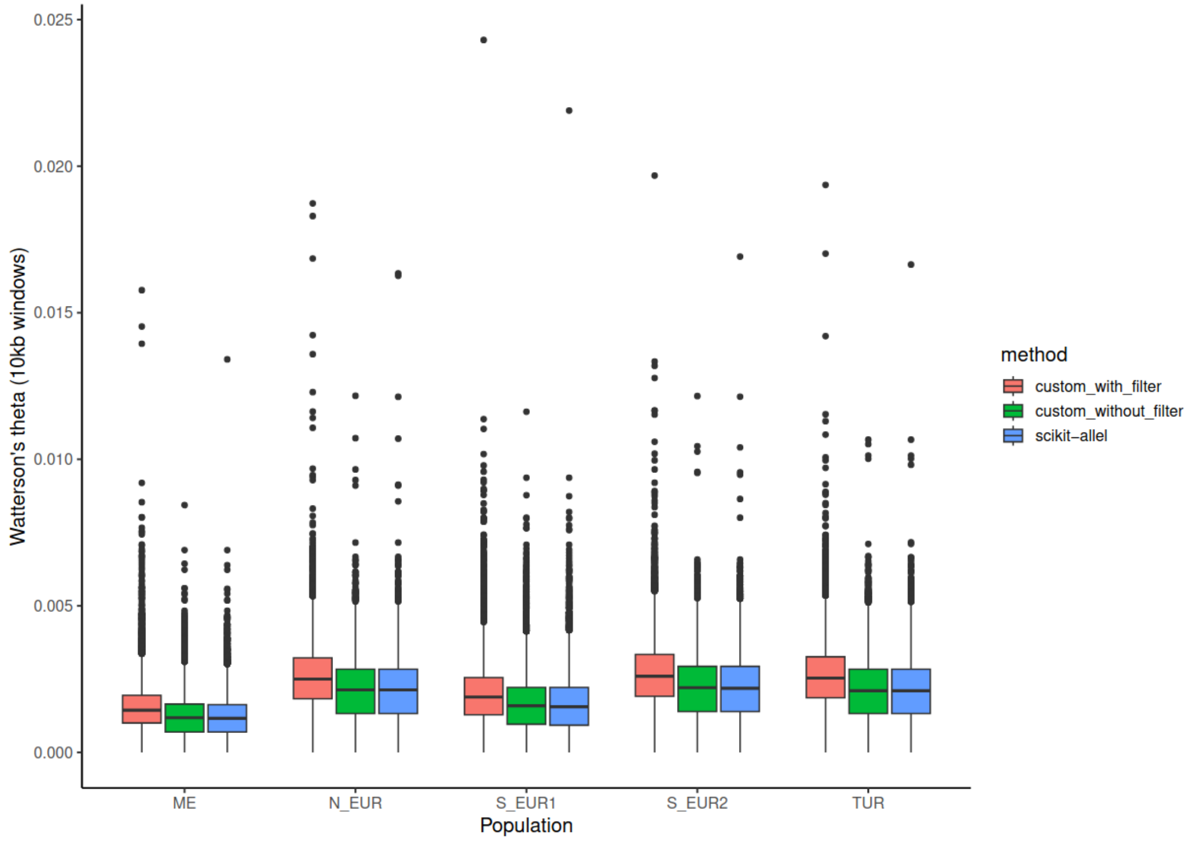

Fig. Watterson's theta estimates using different methods. '*custom-with-filter*' represents our original method, '**custom-without-filter**' is when we do not filter out sites without valid genotypes, and '**scikit-allel**' uses the "allel.windowed_watterson_theta" function in scikit-allel.

Other comments:

8) L133: the « homogeneity » of the Northern European population is not obvious from S4 and S5 figs. Moreover, the population genetic meaning of « homogeneous » is unclear. Does it mean low diversity? does it mean that most of the variation segregates within populations?

In the original manuscript we did not define genetic "homogeneity" clearly. What we mean is a lack of strong (sub)structure within a population (in this case, the Northern European

population). We now write this explicitly in the manuscript (Lines 201-203). This lack of substructure can be seen in the PCA (S5 Fig) and in the fineSTRUCTURE analysis (S6 Fig).

9) L135: S6 Fig does not show a measure of « relatedness ». In population genetics, relatedness is a measure of the degree of kinship. If the authors want to comment on population size, they should use a proxy for the population size, such as an estimate of the population mutation rate theta.

We modified the manuscript. In the revised version we do not interpret the coancestry matrix inferred by fineSTRUCTURE as an estimate of relatedness. Furthermore, in the revised version we refrain from making conclusions about the population size based on the coancestry matrix. (Lines 206-210, captions Fig 1, S6 Fig, S7 Fig)

Reviewer #2:

I have reviewed the manuscript "Population genomics and molecular epidemiology of wheat powdery mildew in Europe" by J. Jigisha and colleagues. Overall, I found this work to be well-structured, addressing clear and biologically important questions with appropriately designed and well-executed analyses. I praise the authors for their careful and comprehensive approach, particularly in documenting the methodology: the analyses and workflows are described in sufficient detail, and all data has been made available. I have some comments and questions that I hope will help improve the quality of this manuscript.

Comments:

10) I found that the introduction lays out some very relevant questions such as: "How far can a pathogen disperse in one season?" "Which are the main directions and periods of dispersal?" "How many cycles of sexual and asexual reproduction occur each year?" "Where was the origin of the inoculum initiating a disease outbreak in a field?" "How connected are epidemics in different regions?" (lines 73 - 77). As well as: "1) how large-scale population genomics and molecular epidemiology have become feasible for agricultural pathogens, 2) that they can generate insights about their basic biology, and 3)

that they can also provide valuable information for control strategies" (lines 105 - 107). However, due to their complexity and particularity, most of these questions are still unanswered or at most partially answered within the bounds of the wheat powdery mildew in Europe. I propose that the authors revisit the phrasing, so there is a better logic and balance between the main challenges on the field and what they have effectively achieved with their work.

We agree with the reviewer, and we modified the introduction to clarify which are the relevant questions in the field, and what is the specific contribution of this work to answer such questions. Specifically, we added the following paragraph at the end of the introduction:

"We identified several distinct populations of wheat powdery mildew, highlighting a heterogenous landscape of gene flow. We found that the population structure and the gradients of diversity can be largely predicted by wind connectivity, suggesting that wind is an important factor shaping Bgt populations in Europe. We also discovered several loci that were under positive selection in the recent past and reconstructed the evolutionary history of one such locus, coding for the effector AvrPm17, which is recognized by the wheat resistance receptor Pm17." (Lines 158-165)

11) Lines 118-121: The last sentence of this paragraph already describes the population structure. I suggest starting the next section ("Population Structure in Europe and the Mediterranean") with this description for better flow.

We have tested the suggestion of the reviewer; however, we find that the original version has a better flow, and we decided to not change this part of the manuscript.

12) Figure S2: I did not observe the placement of the Australian samples in the PCA. I assume they are "behind" the USA isolates, as stated in S1 Appendix that they are "indistinguishable from the USA isolates." If this is the case, I suggest plotting some of the Australian points on top to make their placement clear.

Indeed, the Australian isolates were hidden behind the USA isolates. We have now modified S2 Fig such that the Australian points are visible.

13) Line 126: While it is evident that geography plays a fundamental role in the population structure, the statement that "all analyses separated the Bgt isolates into five groups" is not entirely accurate. For instance, in the admixture plots, Southern European individuals are differentiated; however, in the PCA analyses, they appear to overlap with other groups.

The different methods used to analyze population structure grouped the Bgt isolates into various levels of hierarchical clusters. When we compare all methods, we find that individuals from the same geographic region are more similar than those from different regions. The five groups that are recognized by all three analyses are: Northern Europe, Southern Europe, Turkey, Israel, and Egypt. However, we have modified this part in the manuscript such that we emphasize less on the number of groups, and more on the broad, overall patterns (Lines 192-197). The differences in the amount of substructure present within each group are highlighted later in the section. (Lines 201-206)

14) Lines 140-141: I believe there is an overinterpretation of the admixture analyses. The authors state: "...While the analysis of population structure could distinguish different populations, we also found evidence for gene flow between them." I disagree. Analyses such as Admixture or fineSTRUCTURE aim to optimize Hardy-Weinberg equilibrium to identify populations in an unsupervised manner. While the coexistence of different ancestry components, or "intermediate genotypes," as described by the authors, may suggest potential gene flow, this requires formal testing. Patterns like these can be explained solely by demography.

We agree with the reviewer, and we were not clear enough in the original manuscript.

In the revised version we do not interpret admixture and shared ancestry as evidence for gene flow (Lines 212-216). Moreover, we added the following sentence to further emphasize this point:

“Moreover, it was shown before that patterns of shared ancestry can also be generated by sampling biases or demographic processes that do not include admixture (Lawson et al 2018)”. (Lines 222-224)

Please see also our answer to comment n. 2 by reviewer 1.

15) Lines 164-187: I feel there is a lack of a clear hypothesis in the "Demographic inference" section. It should come as no surprise that pathogenic fungi exhibit high variation in reproductive success, making models that account for this, such as the Beta coalescent, more appropriate than the classic Kingman coalescent. While I appreciate the emphasis on the importance of such models, it remains unclear what we have learned from their application to the demographic history of powdery mildew in Europe. Although the analyses indicate "evidence for no or minimal population growth," the observed excess of rare variants across all analyzed populations still requires further interpretation.

The main hypothesis of our demographic analyses is that Bgt populations are characterized by a large variance in reproductive success in Europe. It is true that this is not surprising for crop pathogens, but this is the first time that it was established with a formal statistical analysis. The relevance of these results should not be underestimated, as they highlight the need to consider processes leading to variable reproductive success, such as sweepstake reproduction and recurrent selective sweeps. In addition, our findings imply that all results based on the Kingman coalescent published in the past should be reconsidered. As we wrote in the discussion, potentially these results extend to other crop pathogens, and they might be of relevance for multiple agricultural diseases.

Finally, our demographic analyses demonstrate that the observed excess of rare variants in Bgt populations is due to the large variance in reproductive success, and not to population growth. Therefore, we disagree that this observation needs further interpretation. The large variance in reproductive success can fully account for this. What is still unknown is the cause of such variance in reproduce success. As we wrote in the discussion, to tackle this question we need further theoretical advances.

We modified the manuscript to clarify these points. (Lines 257-260, 276-278)

16) Linked to the previous comment, the authors need to justify the selection of the two populations, N_EUR_2 and E_EUR_2, and show how the SFS (including Tajima's D values) appears for these chosen subpopulations. I wonder whether the results would hold if the sister subpopulation, N_EUR_1, or the entire N_EUR population were chosen instead. Finally, for completeness, the authors may want to consider running the scenario where N_EUR_2 allows for misspecification of the ancestral alleles.

For this analysis we selected populations belonging to the finest classification level identified by fineSTRUCTURE to minimize the confounding effect of population structure. We chose populations N_EUR2 and E_EUR2 because they have the largest number of samples.

We have now expanded this analysis to include the four populations with the largest samples sizes: N_EUR1, N_EUR2, E_EUR1, and E_EUR2. In the revised manuscript we also performed the analysis allowing for the misspecification of ancestral alleles for the four populations. In all (eight) analyses we found overwhelming evidence in favor of the Beta coalescent, supporting our original results. These results are shown in S2 table.

The SFS and Tajima's D values for these four populations are reported in S12 Fig and S2 Table.

17) Lines 246-280: The way this section is written weakens the manuscript. It begins by justifying the sampling strategy of two consecutive years as a means to "investigate the spatial epidemiology." However, after just one paragraph, the authors acknowledge that "with samples from two consecutive years we could not observe a geographic shift in any direction, possibly because of the short time between the two sampling seasons". Given the short duration between sampling events, the lack of a spatio-temporal signal is not unexpected. I suggest that the authors frame the dataset more appropriately, emphasizing that both the older collection and the two-year collection together provide a suitable basis for addressing this epidemiological question.

Thank you for this suggestion, we have now modified the manuscript to emphasize how the temporal variation in our data allows us to explore the spatial epidemiology of powdery mildew at both short and long timescales. (Lines 359-367, 393-397)

18) Lines 313-314: The presence of gene duplications presents challenges when assessing single haplotypes due to the potential for chimeric combinations. In theory, variant "J" could represent a chimeric mixture of variants "A" and "B." Please ensure that this issue is not affecting your results, particularly for variants with low frequencies.

We disentangled the haplotypes of both copies of *AvrPm17* by phasing SNP calls with WhatsHap. It was reported before that WhatsHap has an error rate < 0.2% when using short reads (Patterson et al 2015). Moreover, because *AvrPm17* is a small gene (< 400 bp), and our dataset consisted of paired end reads (2x150bp), we could phase all haplotypes. Finally, in our manuscript, we focus only on the variants found in 12 or more isolates, i.e., we refrain from making any conclusions about the variants with low frequencies.

19) Discussion: It seems natural to discuss the presence of mountain regions, such as the Alps, as a natural barrier that could restrict gene flow in Southern Europe. Did the authors consider this factor in their analysis?

We agree that the Alps and Pyrenees constitute natural barriers to gene flow. Specifically, high mountain ranges are an obstacle to wind circulation, and locations on opposite sides of a mountain range are scarcely connected by wind. While we do not account for altitude directly in our analyses, we capture the effect of mountains on wind circulation in our windscape analysis.

We added the following sentence to the Discussion:

“Furthermore, the results of the population structure analyses and of FEEMS suggest that the two main European mountain ranges, the Alps and Pyrenees, also constitute natural barriers to dispersal, likely due to the lack of host, and to reduced wind connectivity between the two sides of these mountain ranges.” (Lines 559-563)

20) Lines 546-551: The authors mention that their SNP filtering strategy was driven by the distribution of their metrics. After inspecting the QD distribution, it is evident that most values range between 20 and 40, yet the filter was set to 10. As a result, this hard filter allows many poor-quality SNPs (with QD values between 10 and 20) into the dataset. A similar issue arises with SNPs having Mapping Quality values < 45. Can the authors provide evidence that the inclusion of these SNPs does not significantly impact the analyses?

Our strategy for site-based filtering was informed by the distribution of the quality metrics as well as by GATK's recommendations (now added to Methods, lines 770-771) which suggest users to take a lenient approach to hard filtering as each quality metric is evaluated individually and may not reflect the overall quality of the site. We employed stringent filters in downstream steps, for example >90% support for a call and filters on proportion of missing data at a site, ensuring that we used only high-quality SNPs in our analyses.

To show that our results are robust to different site-filtering criteria, we repeated the principal component analysis (PCA) on the *Europe+* dataset with stricter site-based filtering (QD<20 and/or MQ<55) as suggested by the reviewer. This resulted in the exclusion of 334,089 SNPs from the original dataset of 1,683,143 biallelic SNPs. The results of this PCA are extremely similar to those reported previously. We thus conclude that the different filtering strategies do not significantly impact our analysis. We now include this additional analysis in our manuscript (Line 810-815, S5 Figure).

Minor comments:

21) Lines 61 - 63: Please provide citations.

We added the following references (Line 101)

Robishaw, J. D., Alter, S. M., Solano, J. J., Shih, R. D., DeMets, D. L., Maki, D. G., & Hennekens, C. H. (2021). Genomic surveillance to combat COVID-19: challenges and opportunities. *The Lancet Microbe*, *2*(9), e481-e484.

Cardona-Ospina, J. A., Rojas-Gallardo, D. M., Garzón-Castaño, S. C., Jiménez-Posada, E. V., & Rodríguez-Morales, A. J. (2021). Phylodynamic analysis in the understanding of the current COVID-19 pandemic and its utility in vaccine and antiviral design and assessment. *Human Vaccines & Immunotherapeutics*, *17*(8), 2437-2444.

Attwood, S. W., Hill, S. C., Aanensen, D. M., Connor, T. R., & Pybus, O. G. (2022). Phylogenetic and phylodynamic approaches to understanding and combating the early SARS-CoV-2 pandemic. *Nature Reviews Genetics*, *23*(9), 547-562.

22) SFig20: Consider changing the number of bins in the histogram as it may help to visualise the coverage ratio for those samples where the values are around 2X.

We increased the number of bins in the histogram in S20 Fig.

23) Line 739: Typo. It should be "two copies" instead of "to copies".

Thank you, we corrected the typo.
